# Preparation of nanoporous alumina hollow spheres with a highly ordered hole arrangement

**DOI:** 10.1039/c7ra12340j

**Published:** 2018-01-09

**Authors:** Takashi Yanagishita, Masahiko Imaizumi, Toshiaki Kondo, Hideki Masuda

**Affiliations:** Department of Applied Chemistry, Tokyo Metropolitan University 1-1 Minamiosawa, Hachioji Tokyo 192-0397 Japan yanagish@tmu.ac.jp

## Abstract

Nanoporous alumina spheres with an ordered hole arrangement were prepared through a two-step anodization of small Al particles. The hole periodicity in the ordered anodic porous alumina could be controlled by adjusting the anodizing conditions. Nanoporous hollow spheres were also obtained by removal of residual Al in an etchant. Additionally, nanoporous spheres loaded with Au nanoparticles on their surfaces were obtained through electrochemical or chemical deposition of Au nanoparticles. The obtained Au/alumina composite hollow spheres were used as a substrate for surface-enhanced Raman scattering measurements.

## Abstract 2

Nanoporous alumina hollow spheres with a highly ordered hole arrangement prepared by two-step anodization of small Al particles.

## Graphical abstract

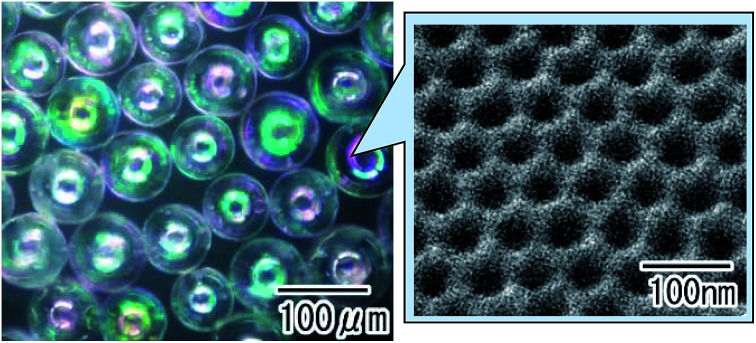

## Introduction

There has been increasing interest in nanoporous particles and hollow spheres because of their potential application in several types of functional devices, such as catalysts, sensors, and drug carriers.^1–9^ To optimize the properties of nanoporous particles towards specific applications, control of the porous structure at the surface of the particles is essential. Various techniques for the preparation of nanoporous particles have been reported so far, including template synthesis, self-assembly processes, and electrospraying.^10–16^ However, precise control of the geometrical structures in the porous layer (*i.e.*, hole diameter, hole periodicity, and hole depth) is usually difficult in these processes. Previously, we reported a new type of process for the preparation of nanoporous particles or hollow spheres based on the anodization of small metal particles.^17,18^ Anodization is a versatile method for the formation of porous oxide films on the surface of valve metal substrates including Al, Ti, Ta, and W.^19–21^ In our reported process, the anodization of small metal particles (Al, Ti) was carried out using closed-packed small metal particles, in which each particle was electrically connected through the contact points. After the anodization, hollow spheres could also be obtained by dissolving the residual metal in an appropriate etchant. The geometrical structures in the obtained porous oxide layer could be controlled using the anodizing conditions. However, the degree of ordering in the hole arrangement and the size uniformity of the holes in the porous layer prepared by this process were low because the holes were developed randomly at the initial stage of the anodization. In the present report, we describe the preparation of nanoporous particles and hollow spheres with a highly ordered hole arrangement. This was achieved by a two-step anodization process in which an ordered concave array formed on the Al surface after the removal of the oxide layer prepared in the first anodization generates an ordered hole array structure during the second anodization.^22^ This work is the first report in which a highly ordered hole arrangement has been achieved during the anodization of small Al particles. The preparation of composite nanoporous spheres loaded with Au nanoparticles, and their application as a substrate for surface-enhanced Raman scattering (SERS) measurements originating from localized surface plasmon resonance, was also investigated.

## Experimental

**Fig. 1:**
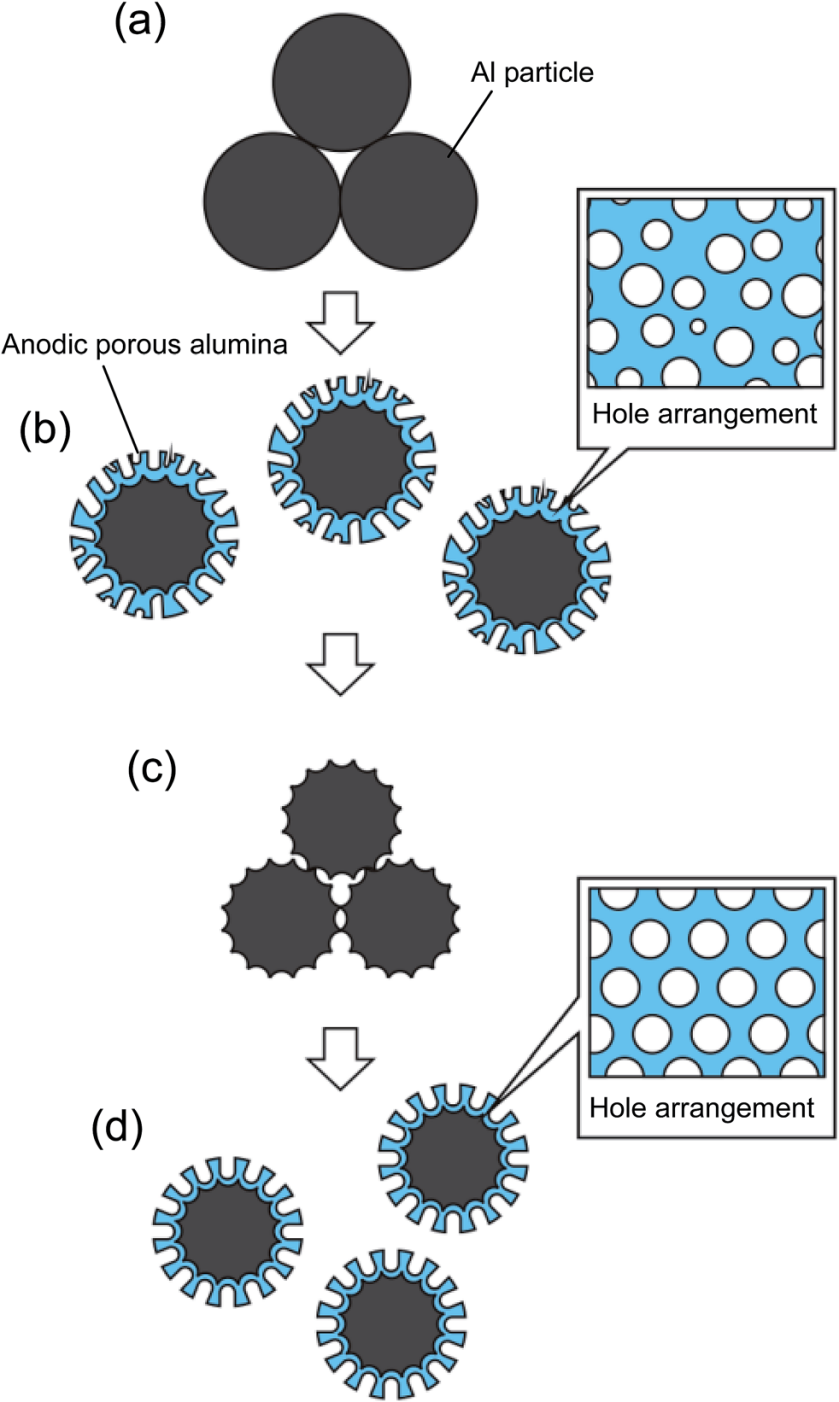
Illustration of the process used to prepare nanoporous spheres with an ordered hole arrangement by two-step anodization of Al particles; (a) packing of Al particles, (b) first anodization, (c) removal of oxide layer and packing of Al particles, and (d) second anodization.

Fig. 1 shows a schematic illustration of the process used to prepare ordered porous alumina spheres by the two-step anodization of Al particles. In this study, spherical Al particles (with an average diameter of 96 μm and a purity of 99.99%, Fukuda Metal Foil and Powder Co., Japan) were used as a starting material. Prior to the anodization, the Al particles were etched using a mixed solution of 1.8 wt% chromic acid and 6 wt% phosphoric acid to remove the native oxide layer on their surfaces. The pretreated Al particles (1 g) were packed into a cylindrical holder made from nylon mesh with a hole size of 75 μm. A cylindrical Al electrode was then set on top of the packed Al particles under an appropriate pressure, and was held in position during the anodization process to ensure the electrical connection between the Al particles. Pressure was applied by a mass of 5 kg cm^−2^. The two-step anodization process reported previously was adopted to prepare ordered anodic porous alumina on the surface of the Al particles.^22^ In this process, a first long period of anodization and subsequent removal of the oxide layer formed in the first period of anodization generated ordered concave arrays on the surface of the Al substrate. The ordered anodic porous alumina structure was obtained by a second anodization of the textured Al substrate using the same anodizing conditions as for the first anodization; the shallow concave structures formed in the first period of anodization acted as starting points for the development of holes. In the present experiments, the first anodization of the Al particles was carried out in a 0.3 M sulfuric acid solution under a constant voltage of 25 V at 17 °C for 60 min.^23^ The oxide layer formed by the first anodization was dissolved in a mixed solution of chromic acid and phosphoric acid. After the etching, the Al particles were rinsed in distilled water and then repacked into the cylindrical holder for the second anodization. The second anodization of the Al particles was performed for 2 min, using the same conditions as for the first anodization.

**Fig. 2:**
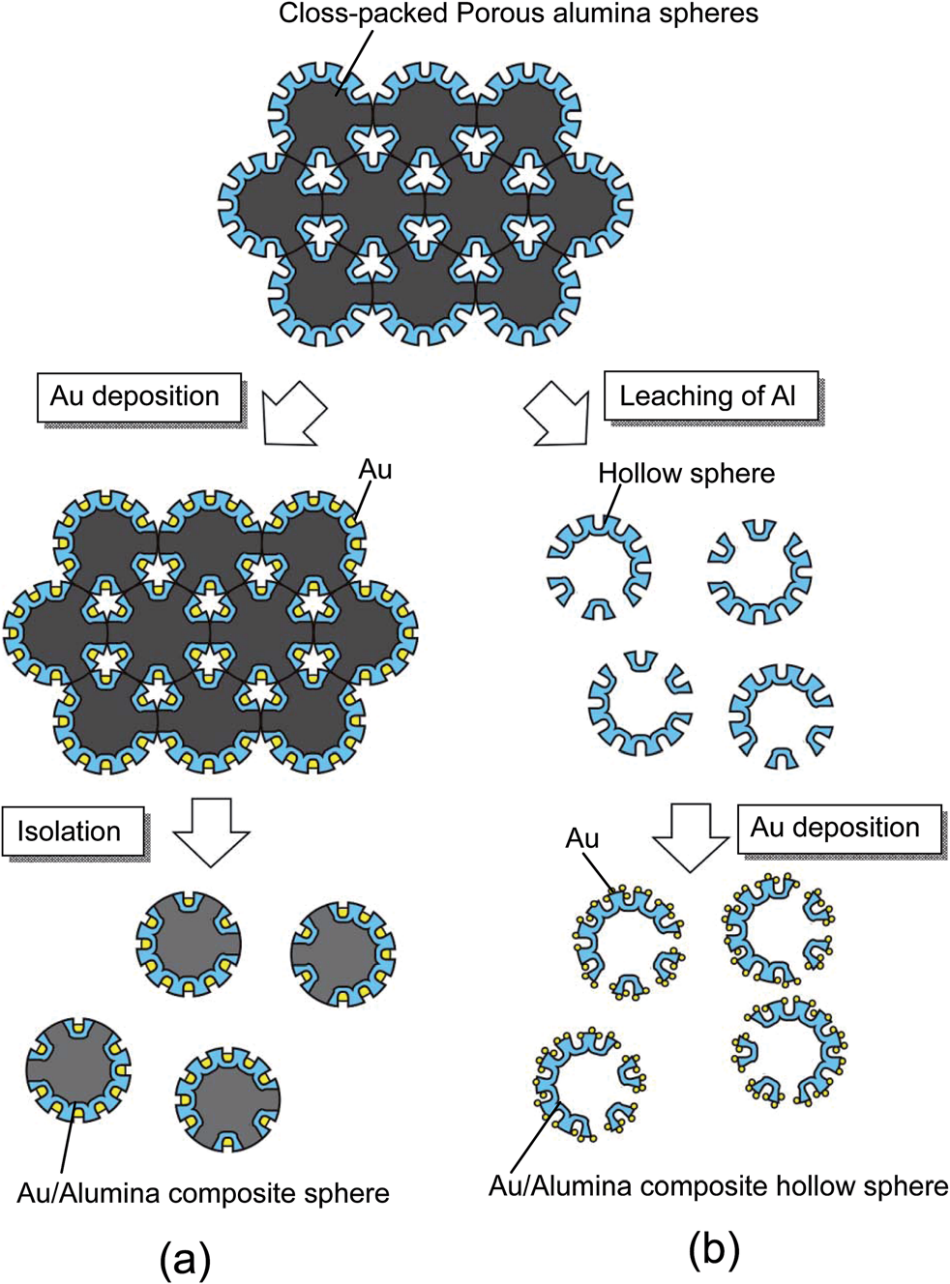
Illustration of the process used to prepare composite spheres loaded with Au nanoparticles on their surfaces; (a) Au/alumina composite spheres formed by electrolytic deposition of Au, and (b) Au/alumina composite hollow spheres formed by chemical deposition of Au.

Fig. 2 shows a schematic illustration of the processes used to prepare two types of composite spheres loaded with Au nanoparticles: Au/alumina composite spheres are shown in Fig. 2(a), and Au/alumina composite hollow spheres are shown in Fig. 2(b).

Au/alumina composite spheres were prepared by the electrolytic deposition of Au into the holes of the anodic porous alumina, using an electrolyte containing 1 g L^−1^ of HAuCl_4_ and 7 g L^−1^ of H_2_SO_4_. After the anodization, the Al particles were rinsed with distilled water and immersed in the Au plating solution while still packed in the holder to maintain their packing structure. The Au electrodeposition was carried out using alternating current (AC) electrolysis, which was performed at 15 V for 5 min. The Au/alumina composite spheres were finally obtained by rinsing with distilled water and drying in air.

For the preparation of the Au/alumina composite hollow spheres, the residual Al in the anodized Al particles was dissolved by immersing the particles in a saturated iodine methanol solution at 50 °C for 12 h. In this process, the contacting portions of the Al particles were not anodized because it was difficult for the electrolyte to penetrate into these areas. Residual Al could leach out from the un-anodized parts of the particles. The formation of Au nanoparticles on the hollow spheres was carried out through chemical deposition using an aqueous solution containing 3.3 mM HAuCl_4_, which was adjusted to pH 7 using NaOH.^24^ The alumina hollow spheres were immersed in the solution at 70 °C for 24 h, and then washed with distilled water. The samples were then annealed at 300 °C for 1 h to form Au nanoparticles on the surface of the alumina hollow spheres. The obtained samples were observed using a scanning electron microscope (SEM; JEOL JSM-6700F). SERS measurements were carried out using Raman spectroscopic apparatus (JASCO NRS-2000), using an He–Ne laser for excitation.

## Results and discussion

**Fig. 3:**
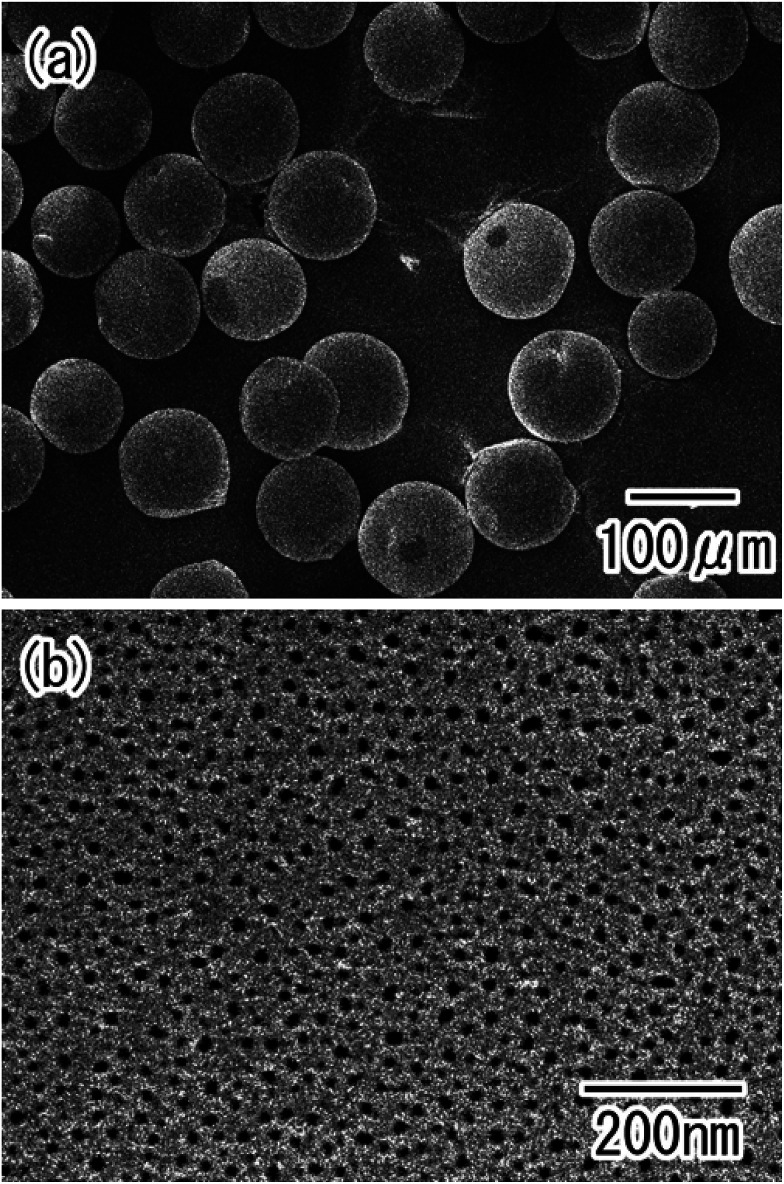
SEM images of the Al particles after the first anodization; (a) low-magnification, and (b) high-magnification view.

Fig. 3 shows SEM images of the Al particles after the first anodization. After anodization, the particles were easily isolated from each other, as shown in Fig. 3(a). The surface SEM image in Fig. 3(b) shows that anodic porous alumina with a disordered hole arrangement was formed on the surface of the Al particles.

**Fig. 4:**
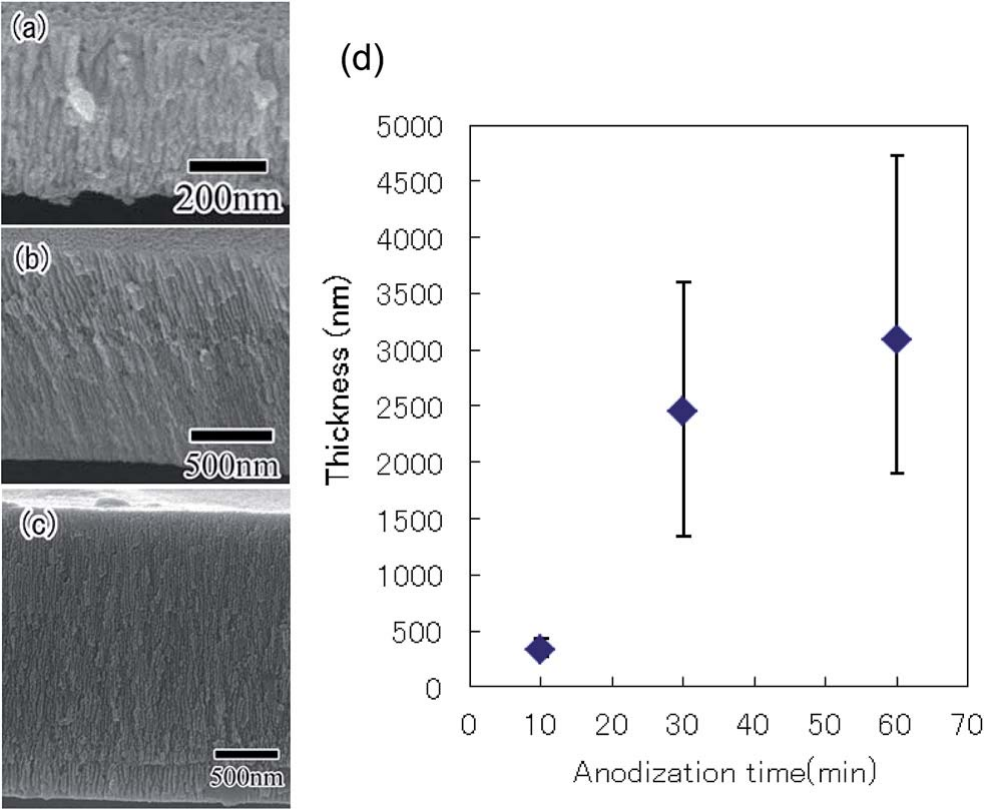
Cross-sectional SEM images of anodic porous alumina obtained by anodization performed for (a) 10, (b) 30, and (c) 60 min. (d) Relationship between anodization time and thickness of porous alumina.

The ordering degree of a hole arrangement in anodic porous alumina depends on the thickness of the oxide layer.^23^ To form a highly ordered anodic porous alumina on the surface of the Al particles, controlling the thickness of the oxide layer was important. Here, thickness of the oxide layer was controlled by changing the anodization time. Fig. 4(a)–(c) show cross-sectional SEM images of the anodic porous alumina with different thickness prepared by anodization for 10, 30, and 60 min, respectively. The SEM images in Fig. 4(a)–(c) confirmed that the thickness of the oxide layer increased with anodization time. Fig. 4(d) shows the relationship between the anodization time and the thickness of the oxide layer formed on the surface of the Al particles. The SEM observations indicated that the variation in the thickness of the oxide layer in each particle was small. However, the variation in thickness between particles was relatively large after 30 min of anodization. We believe that this variation was caused by the experimental setup used for the present study. Particularly, the anodization of the Al particles was stopped when electrical disconnection occurred as a result of movement of the particles in the holder during the anodization process. The variation in thickness could be improved by making improvements to the holder used for the anodization of the Al particles.

**Fig. 5:**
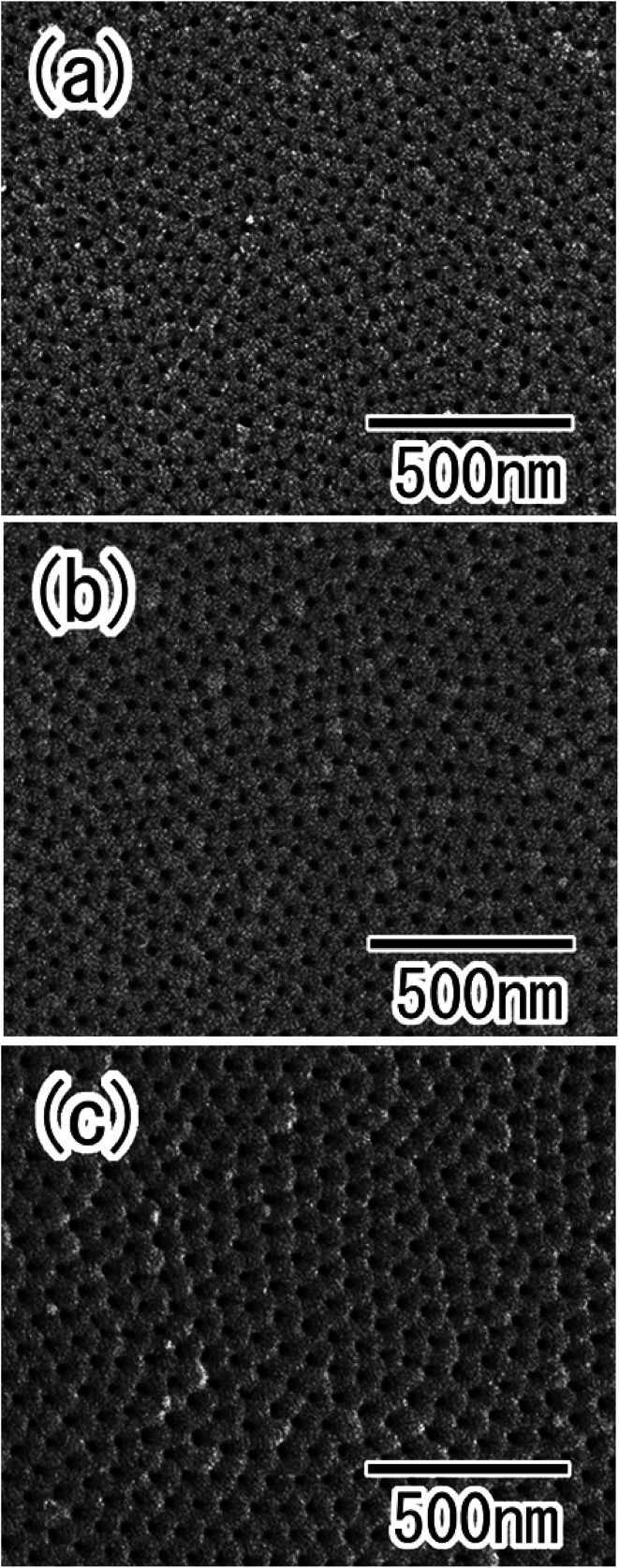
SEM images of anodic porous alumina prepared by the two-step anodization, with first anodization time of (a) 10, (b) 30, and (c) 60 min.

Fig. 5(a)–(c) show SEM images of the anodic porous alumina obtained by the two-step anodization of the Al particles, which was performed for 10, 30, and 60 min, respectively. From the SEM observation of Al particles after etching treatment, it was confirmed that the oxide layer formed by the first anodization was dissolved completely. In our previous works using an Al plate, we reported that the ordered anodic porous alumina could be prepared by long term anodization because the ordering degree of hole arrangement in anodic porous alumina increased with increasing the hole depth, which was dependent on the anodization time.^23,25^ In the case of the anodization of Al particles, the degree of order in the hole arrangement in the anodic porous alumina also increased with anodization time. The hole periodicity of the obtained porous alumina was 63 nm. This value was in good agreement with that observed for anodic porous alumina formed on the surface of an Al sheet.^23^ Using the present process, a large number of nanoporous spheres with an ordered hole arrangement on their surfaces could be obtained simultaneously. In our previous study, it was confirmed that the porous oxide layer could be formed on the surface of Al particles with less than 10 μm in diameter by the anodization process reported here.^17^ This indicates that the preset process can be applied to the preparation of ordered porous alumina spheres with less than several microns in diameter.

**Fig. 6:**
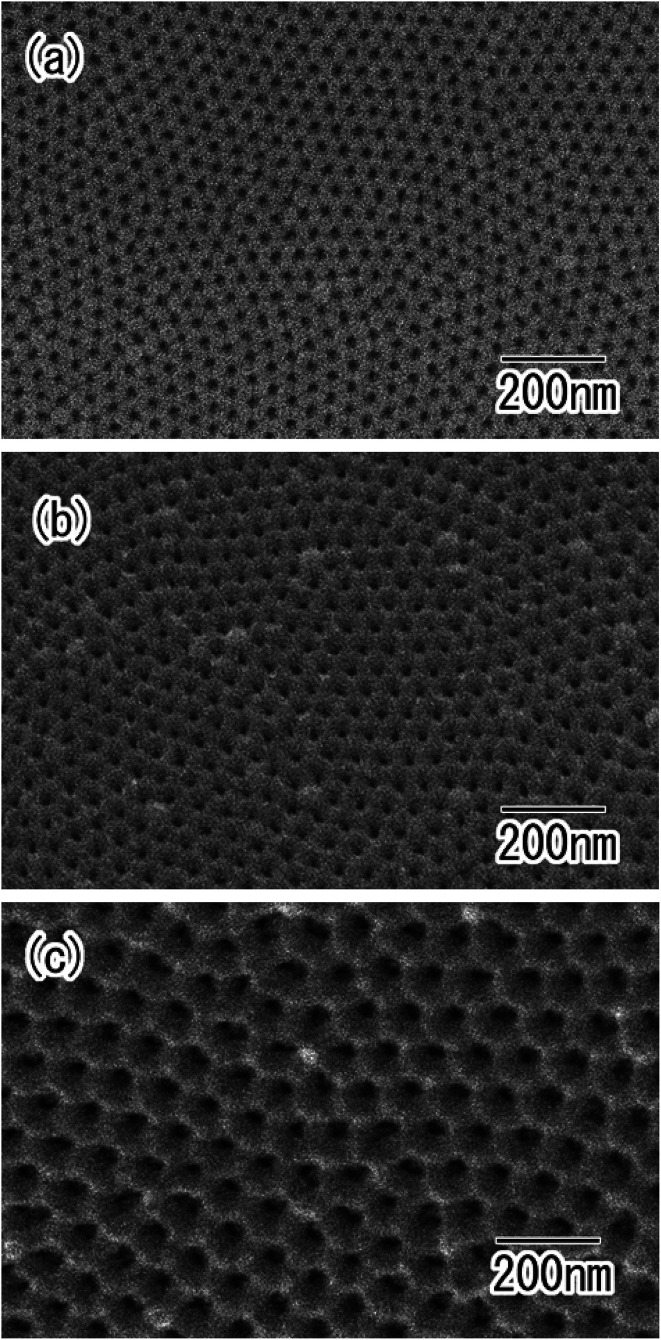
SEM images of ordered anodic porous alumina with different hole periodicity of (a) 45, (b) 63, and (c) 100 nm.

The hole periodicity of the anodic porous alumina could be controlled by adjusting the anodization voltage. Fig. 6(a)–(c) show SEM images of ordered anodic porous alumina with different hole periodicities of 45, 63, and 100 nm, respectively. For these samples, the two-step anodization of the Al particles was carried out under a constant voltage of 18, 25, and 40 V, respectively.^19,23,25^ In all cases, ordered anodic porous alumina was formed on the surface of the Al particles. Form the SEM observation, it was confirmed that the depth of the pore in obtained ordered nanoporous spheres shown in Fig. 6 were 500 nm. The depth of the pore could be controlled by adjusting the anodization time. From these results, we concluded that the present process is useful for the high-throughput preparation of ordered nanoporous spheres with controlled hole periodicity.

**Fig. 7:**
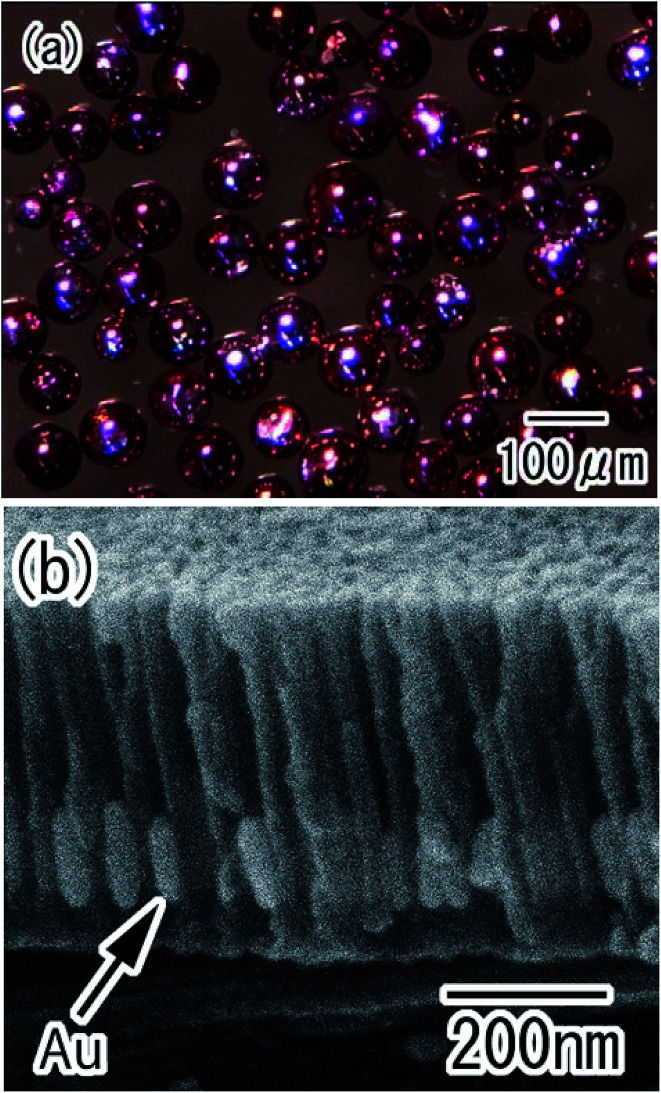
Au/alumina composite spheres obtained by electrolytic deposition of Au nanoparticles into the holes of the alumina layer; (a) optical microscope image, (b) cross-sectional SEM image of the porous alumina layer.

Various metals or semiconductors can be deposited into the holes of anodic porous alumina by electrolytic deposition.^26–28^ The present process was combined with electrolytic deposition to prepare composite spheres with nanoholes loaded with different nanoparticles. Fig. 7 shows Au/alumina composite spheres prepared using AC electrolysis to deposit Au nanoparticles into the alumina holes. After the deposition of the Au, the color of the spheres changed from silver to red, as shown in Fig. 7(a). This color change was caused by the surface plasmon absorption of the Au nanoparticles deposited in the holes of the alumina layer. Fig. 7(b) shows a cross-sectional SEM image of the porous alumina layer after the Au deposition. Uniformly-sized, roughly cylindrical Au nanoparticles were produced in the bottom region of the alumina holes. The average diameter and height of the Au nanoparticles were determined to be 40 and 110 nm, respectively. The height of the Au nanoparticles could be controlled by adjusting the electrolysis time.

**Fig. 8:**
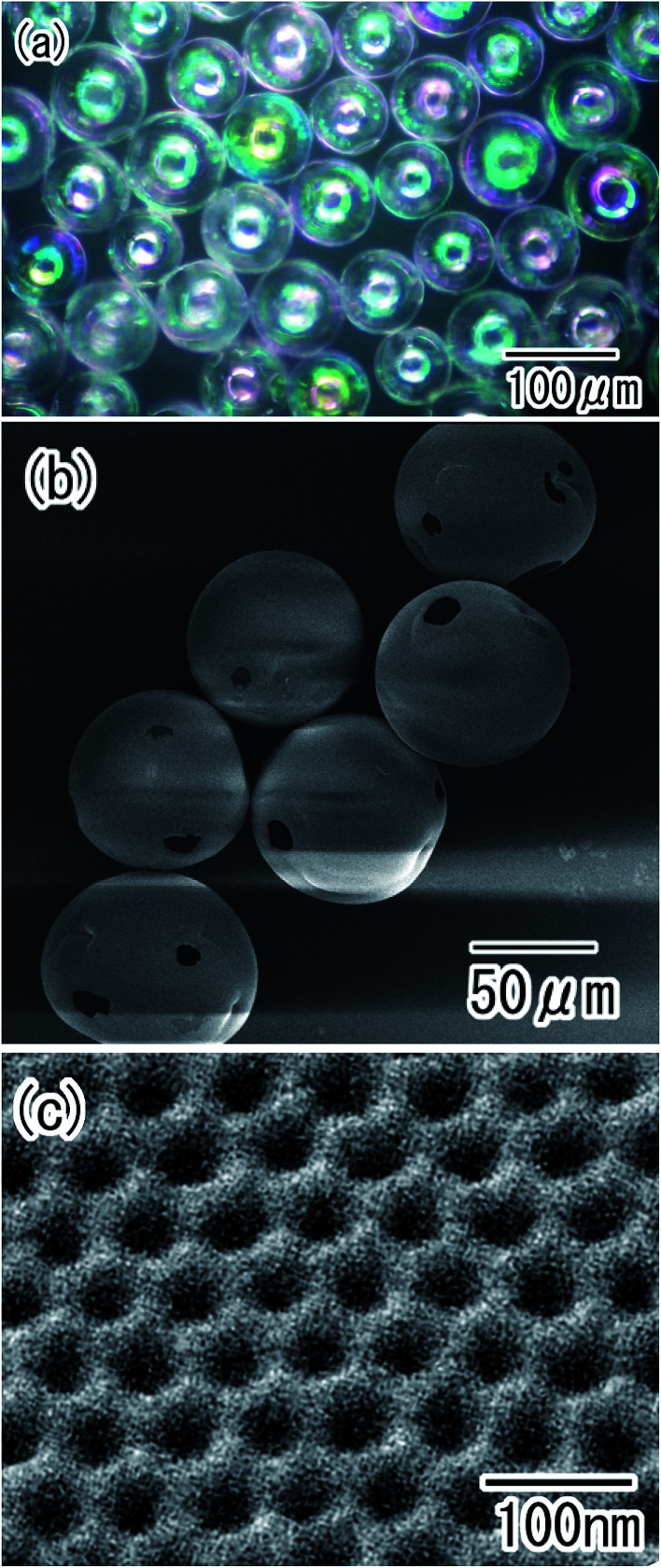
Ordered porous alumina hollow spheres obtained by removal of residual Al; (a) optical microscope image, and (b) low-magnification and (c) high-magnification SEM images.

Porous alumina hollow spheres were also obtained by the removal of residual Al from the particles in an etchant. The optical microscope image shown in Fig. 8(a) confirmed that transparent spheres were obtained. The spherical shape of the Al particles was maintained even after the removal of Al. Micro-sized holes were observed in each sphere, as shown in Fig. 8(b). These holes corresponded to the regions of contact between particles during the anodization process; the residual Al was dissolved from the unoxidized regions of the Al particles in the etchant. The surface SEM images in Fig. 8(c) showed that the shell of the hollow spheres was composed of ordered anodic porous alumina layer with a hole periodicity of 63 nm.

**Fig. 9:**
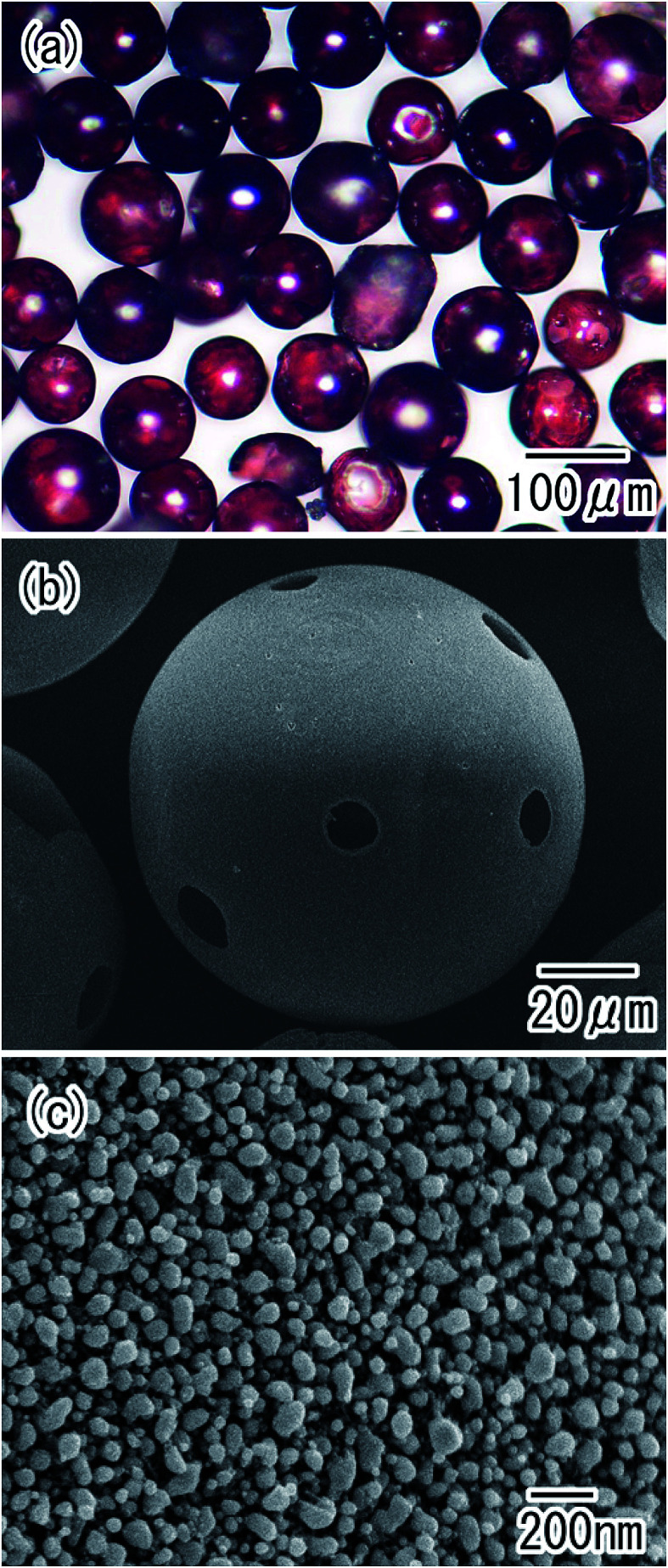
Au/alumina composite hollow spheres obtained by chemical deposition of Au nanoparticles on their surfaces; (a) optical microscope image, and (b) low-magnification and (c) high-magnification SEM images.

Chemical deposition also proved to be an effective technique for the preparation of composite spheres.^29,30^Fig. 9 shows Au/alumina composite hollow spheres formed through the chemical deposition of Au nanoparticles on the surface of the alumina hollow spheres. Transparent red spheres were observed in the optical microscope image shown in Fig. 9(a). The spherical shape of the alumina hollow spheres was maintained after the Au deposition treatment, as shown in Fig. 9(b). Fig. 9(c) shows an SEM image of the surface of the composite hollow spheres. Au nanoparticles with a diameter of 10 to 40 were formed on the surface of the alumina hollow spheres. The preparation of hollow spheres with ordered pores permeating through the whole shell thickness will be prepared by the electrodeposition of a metal into pores and subsequent selective etching of residual Al.

**Fig. 10:**
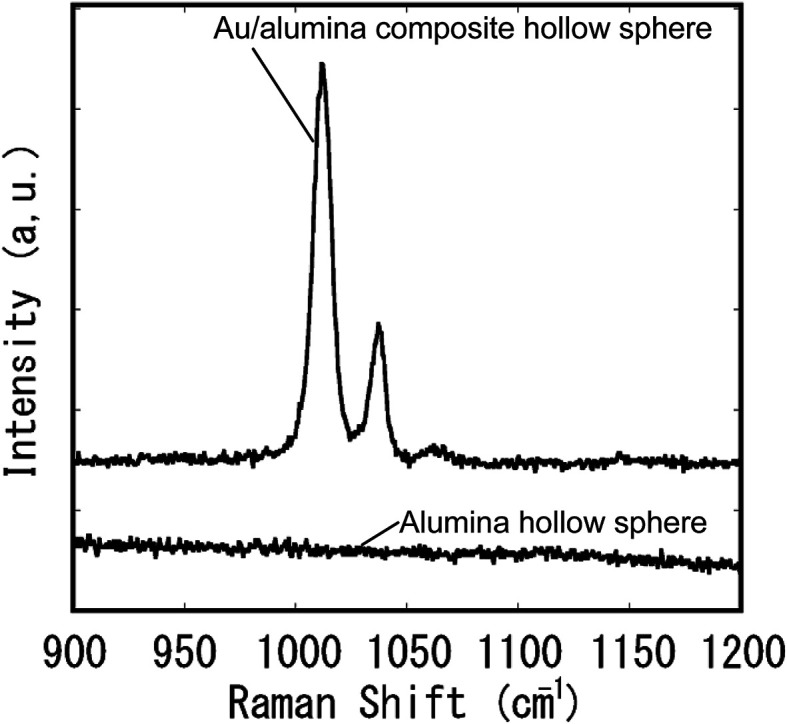
SERS spectra of pyridine.

The obtained Au composite spheres were applied as a substrate for SERS measurements. Fig. 10 shows Raman spectra of pyridine molecules measured using alumina hollow spheres with and without Au nanoparticles on their surfaces. The Raman spectra were measured after dipping the hollow spheres in a solution of pyridine, and drying in air. No signals were observed in the spectrum of the alumina hollow spheres. In contrast, two very large signals corresponding to the pyridine were observed in the spectrum of the Au/alumina composite hollow spheres.^31^ This confirmed that the Au/alumina composite hollow spheres acted as an effective substrate for the SERS measurements. The three-dimensionally assembled composite hollow spheres could also be used as a SERS substrate, owing to their transparency. It is expected that the assembled structures will provide an improved substrate for the realization of ultramicroanalysis because of their large surface area.

## Conclusion

Nanoporous spheres with a hexagonally arranged array of uniform-sized holes on their surface were prepared by two-step anodization of small Al particles. The hole periodicity in the ordered anodic porous alumina formed on the surface of the Al particles was controlled by adjusting the anodization conditions. Composite spheres loaded with Au nanoparticles were also obtained through electrolysis or chemical deposition. The obtained Au/alumina composite hollow spheres were used as a substrate for SERS measurements. The ordered nanoporous spheres and composite spheres obtained using the present process can be used in a variety of applications as catalysts, drug carriers, and SERS substrates.

## Conflicts of interest

There are no conflicts to declare.

## Supplementary Material
